# Premature coronary artery disease in women: sex-specific risk factors, pathogenetic mechanisms and clinical implications

**DOI:** 10.1080/07853890.2026.2613563

**Published:** 2026-01-30

**Authors:** Fei Li, Dan Hong, Mei Yang, Zhilin Pang, Haolin Xi, Qingyuan Zhang, Chuanchang Li, Long Mo, Liming Peng

**Affiliations:** a Department of Geriatric Medicine, Xiangya Hospital, Central South University, Changsha, Hunan, China; b Department of Cardiology, Xiangya Hospital, Central South University, Changsha, Hunan, China; c National Clinical Research Center for Geriatric Disorders (Xiangya Hospital), Central South University, Changsha, Hunan, China

**Keywords:** Premature coronary artery disease (PCAD), sex-specific risk factors, pathogenetic mechanisms, clinical implications, genetic predispositions

## Abstract

**Introduction:**

Premature coronary artery disease (PCAD) in women, defined as onset ≤65 years, accounts for 20–30% of CAD cases. The clinical implications, pathogenic processes, and sex-specific risk factors of female PCAD are summarized in this review.

**Discussion:**

Women have unique patterns in the traditional metabolic risk factors of smoking, diabetes, obesity, and hypertension. Elevated triglyceride-rich lipoproteins and lipoprotein(a) are highly associated with early atherosclerosis. Premature menopause, autoimmune diseases like systemic lupus erythematosus, and pregnancy difficulties like preeclampsia and gestational diabetes are among the special factors that affect women and worsen inflammation and endothelial dysfunction. PCAD progression is also influenced by inflammatory pathways (e.g. NLRP3 inflammasome activation) and genetic predispositions (e.g. 9p21 locus polymorphisms, familial hypercholesterolemia). Diagnostic delays occur because women frequently appear clinically atypically (e.g. fatigue, nausea) and show non-obstructive lesions or spontaneous coronary artery dissection (SCAD). Sex-tailored approaches are needed for management, such as aggressive lipid control, metabolic risk reduction, and psychosocial support.

**Conclusion:**

Female PCAD is a complex condition influenced by a combination of traditional metabolic risk factors, sex-specific factors, and genetic predispositions. Sex-specific biomarkers and multi-omics techniques should be given top priority in future studies in order to improve early detection and tailored treatment.

## KEY MESSAGES

1. Female PCAD patients exhibit distinct metabolic and sex-specific risk factor profiles, necessitating tailored management strategies.
2. Genetic predispositions and sex-specific factors, including premature menopause and autoimmune diseases, significantly contribute to the risk and progression of PCAD in women.
3. Early detection and sex-tailored interventions, incorporating advanced biomarkers and multi-omics techniques, are crucial for improving outcomes in female PCAD.

## Introduction

Cardiovascular diseases (CVD) remain the leading cause of death worldwide. In 2019, 17.1 million deaths worldwide were attributed to CVD, which is a significant increase from 1999 [1]. Over 9 million deaths are predicted to be attributable to coronary artery disease (CAD) by 2030 [2]. The 2023 Report on Cardiovascular Health and Diseases in China states that CVD-related deaths account for 45% of all fatalities. There are 330 million individuals with CVD, including 11.39 million CAD patients [3]. According to recent epidemiological data, 10% of cases of CAD occur in individuals under 45 years of age, even though the condition primarily affects the elderly [4]. People under the age of 55 and 60 make up 17.9% of cases and 12.3% of CAD-related deaths, respectively, according to recent U.S. statistics [5]. Notably, young adults in North America are experiencing an increase in the incidence of myocardial infarction (MI) [6]. Despite major advancements in diagnosis and treatment, cardiovascular disease (CVD) remains a major global cause of morbidity and mortality among young people.

According to the National Cholesterol Education Program Adult Treatment Panel III (NCEP-ATP III) guidelines [7–10], the beginning of premature coronary artery disease (PCAD), which makes up 20%–30% of all cases of CAD, is ≤55 years for men and ≤65 years for women [11]. Women account for 20 to 30% of PCAD cases [7,12,13]. PCAD is a polygenic disorder with a high heritability rate (50–60%) and robust gene-environment interactions. Compared to their non-PCAD counterparts, PCAD patients typically suffer from acute coronary syndrome, which is defined by abrupt disease progression without prodromal symptoms.

Conventional wisdom holds that oestrogen-mediated endothelial protection reduces women’s risk of premenopausal CAD [14,15]. However, new epidemiological data reveal contradicting mortality trends: despite a significant decline in male CAD mortality, female CAD death rates unexpectedly increased [16,17], raising doubts about sex-specific pathophysiological mechanisms. There are distinct sex-based disparities in PCAD’s onset age, physical characteristics, and clinical presentations. Young women have higher incidence rates of MI and worse clinical outcomes, including longer hospital admissions and higher in-hospital mortality [18]. Due to increased professional and family demands brought on by modern culture, PCAD is becoming more prevalent in women. This condition has a significant impact on people, families, and healthcare systems. This review systematically examines the key characteristics and public health consequences of female PCAD across three dimensions: molecular epidemiology, clinical phenotypic heterogeneity, and prognostic inequalities. Crucially, this review addresses not only factors exclusive to women (e.g. pregnancy, menopause) but also traditional cardiovascular risk factors. We include the latter because emerging evidence indicates they often carry a higher relative risk or operate through distinct pathophysiological pathways in young women compared to their male counterparts.

## 1. Traditional risk elements

While established risk factors such as hypertension, dyslipidaemia, diabetes mellitus and smoking affect the general population, they frequently exert a disproportionately heavier burden on young women. It has been discovered that these factors all have a substantial impact on the pathogenesis of PCAD in women, often acting as more potent risk multipliers in females than in males. It has been demonstrated that several of these parameters are especially strongly correlated with mortality [19].

### 1.1. Risk factors for metabolism

According to a systematic review of 41 studies, female PCAD patients had distinct lipid profile abnormalities compared to males. These included higher levels of HDL-C and total cholesterol, as well as significantly higher risks of metabolic syndrome (MetS, OR = 3.73), obesity (OR = 1.33), diabetes (OR = 1.78), and hypertension (OR = 1.51) [9]. Sex-specific lipid risk patterns are evident: The Cardiovascular Risk of Young Finns. The research indicates that if lipoprotein(a) [Lp(a)] ≥ 75 nmol/L (HR = 2.0, 95% CI 1.4–2.6), the risk of atherosclerotic cardiovascular disease (ASCVD) in young women is twofold. For every standard deviation increase in Lp(a), the risk of CAD in women under the age of 55 rises by 22%. Interestingly, triglyceride-rich lipoproteins (TRLs) and their metabolites are more significantly linked to ASCVD than low-density lipoprotein cholesterol (LDL-C) in women under the age of 55 [20]. These findings align with studies on subclinical atherosclerosis, which demonstrate that even in normolipidemic populations (mean age, 45.5 years), hypertriglyceridemia (>150 mg/dL) remains significantly associated with subclinical atherosclerosis and vascular inflammation [21]. This finding suggests that TRLs could be novel biomarkers and therapeutic targets for female PCAD.

### 1.2. Hypertension and type 2 diabetes

Two significant risk factors for CAD include type 2 diabetes (T2D) and high blood pressure. While these are shared risks, their impact is sex-dimorphic. Women with diabetes have a higher risk of PCAD than men [22], and diabetic women face higher mortality rates than their male counterparts [23]. Furthermore, the risk is compounded by female-specific history, such as prior gestational diabetes [24].

Even though prevalence increases with age, young CAD patients exhibited significantly higher rates of diabetes and hypertension than non-CAD populations [25]. Women with PCAD are more likely than men to experience the cumulative cardiovascular effects of these metabolic abnormalities [26,27]. The prevalence of hypertension in female PCAD patients was 69.5%. This susceptibility in women is closely linked to the loss of oestrogen-mediated downregulation of the renin-angiotensin system (RAS), which can lead to rapid blood pressure escalation during the menopausal transition. For every 20 mmHg increase in systolic blood pressure, the risk increases by 60% [28]. Women with diabetes have a 1–4 times higher risk of PCAD than men [29], whereas male patients have a 2–3 times higher risk of mortality than female patients [30]. Interestingly, diabetes is the primary cause of MI in women; women with diabetes are 8 times more likely to get MI than women without diabetes [31].

### 1.3. Obesity

Obesity (BMI ≥ 30 kg/m^2^) in female PCAD has several pathophysiological effects. Unlike in men, obesity in young women frequently interacts with visceral adipose-driven VLDL overproduction to create a more aggressive ‘metabolic toxicity’ phenotype. In addition to conventional metabolic dysregulation, adipose tissue endocrine dysfunction causes proinflammatory cytokine release (e.g. IL-6, TNF-α), increases vascular oxidative stress, polarizes macrophages because of visceral fat accumulation, and inhibits endothelial nitric oxide synthase (eNOS), all of which accelerate endothelial dysfunction and plaque destabilization. Epidemiological statistics show that the prevalence of obesity in PCAD populations is 47.1%, with a distinct age stratification: the subgroup of people aged ≤35 years has much higher obesity rates (53.2%) than the groups aged 35–45 (49.1%) and 45–55 (44.2%). *via* visceral adipose-driven VLDL overproduction, young obese women have exacerbated dyslipidaemia, with LDL-C abnormalities (11.2%) occurring 2–3 times more commonly than in older groups [32]. The YOUNG-MI registry finds that 43.9% of young female CAD patients are obese, whereas Chinese population studies reveal rates as high as 53.7%, which is much higher than their male counterparts [33,34]. These large-scale registries reflect this pattern. This finding emphasizes how the pathogenesis of female PCAD involves sex-specific metabolic toxicity.

### 1.4. History of the family

Family history is one independent risk factor for PCAD in women. Cohort studies show that 64% of young CAD patients have a positive family history [35]. Specifically, 28.5% to 35% of female PCAD patients report having a CAD family history [33,34], which is associated with a 4-fold higher risk of myocardial infarction. Women with a family history of PCAD frequently have clustering metabolic abnormalities: 67% have both hypertension and dyslipidaemia concurrently, 53% are obese, 42% smoke, and 33% have diabetes [36]. ASCVD, PCAD, and diabetic family history are strongly correlated with each other [37].

### 1.5. Hyperuricemia

Hyperuricemia (HUA), which is defined as serum uric acid >360 μmol/L, emerges as a novel metabolic risk factor in female PCAD *via* a unique ‘uric acid-inflammation-vascular injury’ route. Its pathogenicity goes beyond crystalline deposition in three non-crystalline ways: ① Uric acid-mediated reactive oxygen species (ROS) burst impairs endothelial nitric oxide (NO) bioavailability, leading to vasodilatory dysfunction; ② Activation of the NLRP3 inflammasome stimulates the release of proinflammatory cytokines (IL-1β, IL-18), speeding up the development of atherosclerotic plaque; ③ Renin-angiotensin system (RAS) overactivation increases blood pressure (OR = 1.32 per 1 mg/dL UA increase) and insulin resistance (HOMA-IR increase 0.45, *p* < 0.01). Surprisingly, HUA alone is responsible for 17.3% of PCAD risk in nonsmoking women aged ≤35 [38], suggesting that it may be a significant factor in young women’s unusual risk profile. According to U.S. national health surveys, the risk of metabolic syndrome in adolescent females (12–19 years old) is raised by 38% for every 1 mg/dL uric acid elevation, which is significantly higher than the risk for males [39]. These sex-specific analyses indicate that girls have a stronger HUA-PCAD interaction. The equilibrium of urate metabolism is tightly regulated by oestrogen *via* the ABCG2 transporter (ATP-binding cassette super-family G member 2). Physiologically, oestrogen transcriptionally upregulates renal ABCG2 expression, ensuring efficient urate excretion [40]. However, this protective axis is vulnerable: premenopausal hormonal fluctuations and the drastic decline in oestradiol during the menopausal transition lead to a downregulation of ABCG2. This disruption results in a relative hyperuricemia phenotype, where uric acid levels rise rapidly relative to an individual’s baseline, triggering vascular inflammation and oxidative stress more aggressively in women than in men, even if absolute levels remain within normal population limits [41,42]. Large-scale prospective studies that elucidate the dynamic regulatory network of the sex hormone-uric acid axis in PCAD are still lacking.

### 1.6. Smoking

Exposure to tobacco smoke is one of the most potent modifiable cardiovascular risk factors for women. According to recent epidemiological statistics, cardiovascular disease in women is more serious and appears sooner: A Pakistani registry analysis found that 31% of PCAD patients having percutaneous coronary intervention (PCI) were active smokers, compared to 28% of the elderly. Moreover, there was a 28% female predominance among these patients (compared to 10% in the elderly cohort, *p* < 0.001) [43,44]. These results implied a spatiotemporal association between coronary disease rejuvenation and smoking exposure. Framingham data is used to quantify risks unique to sex: Heavy smokers between the ages of 35 and 44 are much more likely to develop peripheral artery disease (PAD) than nonsmokers (male HR = 1.92, female HR = 1.70), and smoking causes women to have their first MI start 8–13 years earlier [45,46]. Nicotine and carbon monoxide cause endothelial dysfunction, dysregulation of lipid metabolism, and inflammation through platelet activation, induction of the procoagulant state, and an imbalance between vasoconstrictor and vasodilator. These factors ultimately lead to thrombogenesis, plaque destabilization, and coronary spasm. Young women (those under 26) are using tobacco and e-cigarettes at high rates. According to nationwide polls conducted in the United States, the use of e-cigarettes has increased by 217% over the last five years. Their formaldehyde and acrolein-containing aerosols induce endothelial pyroptosis and hyperactivation of matrix metalloproteinase-9 (MMP-9) by activating TRPA1 ion channels, which makes plaque more vulnerable than it is with regular smoking [47]. It is possible that this ‘stealth tobacco exposure’ will drastically alter the epidemiological patterns of PCAD in young girls.

### 1.7. Genetic determinants

Like environmental variables, CAD has a substantial hereditary component. Although many genetic risk loci are shared between sexes, their phenotypic expression can be sex-dependent. Understanding these general genetic factors is vital for female PCAD because of their potential interaction with female-specific environmental triggers, such as hormonal fluctuations during pregnancy and menopause. Having first-degree relatives with PCAD, such as parents or siblings, nearly doubles a person’s risk of getting the disease [43]. In the Swedish Twin Registry study (*n* = 21,000, follow-up 35–50 years), heritability estimates for fatal CAD occurrences in males and females were 0.57 and 0.38, respectively, with greater genetic effects observed in younger populations [38]. These findings align with current studies that emphasize the significant contribution of genetics to early CAD [46]. Notably, female PCAD patients have a 15% higher first-degree relative prevalence (35% vs. 30%) and higher familial aggregation compared to males [45]. This finding could suggest that female-specific susceptibility is caused by mitochondrial DNA variants or X-chromosome genes (e.g. coagulation factors VIII/IX) [48,49].

### 2.1. Monogenic contributions

#### 2.1.1. Familial hypercholesterolemia

Familial hypercholesterolemia (FH) is an autosomal dominant condition characterized by elevated low-density lipoprotein cholesterol (LDL-C), which significantly accelerates the development of premature atherosclerotic cardiovascular disease [47]. The carriers of the FH mutation have a 2.6-fold increased risk of developing CAD, which increases to 3.7-fold in PCAD populations, even with subthreshold LDL-C levels (<130 mg/dL) [50]. The primary problematic changes are seen in the genes encoding APOB (apolipoprotein B), LDLR (LDL receptor), and PCSK9 (proprotein convertase subtilisin/kexin type 9) [51,52].

#### 2.1.2. Genes associated with lipid susceptibility

About 40% of PCAD patients suffered from inherited dyslipidaemias [47]. Carriers of the LDLR mutation are ten times more likely to develop early atherosclerosis compared to the general population [48–50]. Low α-lipoprotein raises the risk of early-onset CAD and stroke. Hypoalphalipoproteinemia (HDL-*C* < 40 mg/dL), which is 20% more prevalent in women, is another significant risk factor for CAD and could be linked to oestrogen-mediated reverse transfer of cholesterol through ABC transporter activation. The postmenopausal oestrogen drop accelerates the production of plaque and results in HDL dysfunction (beyond quantitative reduction) [53,54].

#### 2.1.3. Homocysteine metabolism disorders

Hyperhomocysteinemia (HHcy) is an independent atherosclerosis risk factor with sex-specific consequences that are mostly caused by mutations in MTHFR C677T and CBS [55–57]. Due to gender-dimorphic regulation of folate metabolism, females with MTHFR C677T mutations had a 1.5-fold increased risk of PCAD compared to males (OR = 2.3 vs. 1.5) [58,59]. Additionally, HHcy increases the risk of long-term CAD through metabolic memory pathways by increasing the risk of preeclampsia during pregnancy [60].

### 2.2. Polygen-interaction studies

The genetic architecture of CAD involves complex interactions between monogenic and polygenic factors [61]. Genome-wide association studies (GWAS) have shown that the 9p21 locus is the most significant polygenic risk locus, where risk alleles (like rs1333049) regulate CDKN2A/B to promote inflammation and vascular smooth muscle proliferation [49,62,63]. Homozygous carriers of 9p21 polymorphisms have twice the risk of PCAD, with population-attributable risks of 21% for myocardial infarction and 31% for premature occurrences [62]. rs4977574 polymorphism in the CDKN2B-AS1 gene (9p21.3) is notable for its sex-specific effects, and females with allele G are significantly more likely to develop PCAD [64].

#### 2.2.1. Clonal haematopoiesis of indeterminate potential (CHIP)

Early atherosclerosis is associated with CHIP, an aging-related condition in women under 50, characterized by somatic mutations in hematopoietic stem cells (HSCs) that confer leukemogenic potential [65–68]. Between the ages of 45 and 50, CHIP carriers are four times more likely to experience acute MI, which could be induced by elevated inflammation [68]. However, a cohort study (*n* = 200,453) found no significant association with ischemic cardiovascular events (CAD/stroke), raising questions about CHIP’s impact on the cardiovascular system [69].

#### 2.2.2. Gene variants linked to damage to the endothelium

Renin-angiotensin-aldosterone system (RAAS) gene polymorphisms are linked to in-stent restenosis in the Russian population: The A allele of AT2R rs1403543 is a risk factor for restenosis in people under 65, the heterozygote of REN rs41317140 and CYP11B2 rs1799998 is a risk factor for late restenosis, the A allele of REN rs2368564 and the T allele of AGT rs699 are risk factors, and the heterozygote of AGT rs4762 is a protective factor [70].

#### 2.2.3. Inflammation-related genetic variants

Inflammation is a key factor in modulating the progression of CAD from the beginning to plaque destabilization [53,54,59]. The concentration of monocyte chemoattractant protein-1 (MCP-1) is substantially higher in patients with the AA genotype of the rs1024611 polymorphism than in those with the GA/GG genotype. Furthermore, the risk of early-onset coronary heart disease rises by 2.7% in the female population for every 10 pg/mL increase in this concentration, regardless of age or other variables [71]. By controlling the release of IL-6, IL-12, IL-23, and TNF-α from monocytes and macrophages, interferon regulatory factor 5 (IRF5) affects the stability of atherosclerotic plaques [72]. A type B scavenger receptor protein called CD36 can influence how cardiomyocytes absorb long-chain fatty acids, which can result in insulin resistance and problems with lipid metabolism. According to research [73], the Han Chinese population in northern China has extreme lipid profiles and a high risk of PCAD due to four single-nucleotide polymorphisms (SNPs) in the CD36 gene. These SNPs may be a novel target for the treatment of dyslipidaemia.

## 3. Sex-specific risk factors

Female-specific risk factors are not merely statistical associations but are active drivers of PCAD pathology. Unlike traditional metabolic risks, these factors, including pregnancy complications, hormonal fluctuations, and autoimmune conditions, accelerate disease progression by directly fuelling chronic inflammation, promoting endothelial dysfunction, and inducing unique metabolic phenotypes.

### 3.1. Issues concurrent with pregnancy

The incidence of MI during pregnancy was 1 in 35,700 deliveries, with rising trends, according to California statistics from 1991 to 2000 [74]. Maternal mortality reached 7.3% as a result, and an independent predictor was advanced maternal age. These events are generally brought on by thromboembolism, microvascular dysfunction, or SCAD, not atherosclerosis [75–78]. Notably, pregnancy-induced elevation of LDL-C exacerbates atherogenesis in patients with familial hypercholesterolemia, particularly when lipid-lowering medication is discontinued [75].

**Figure 1.**
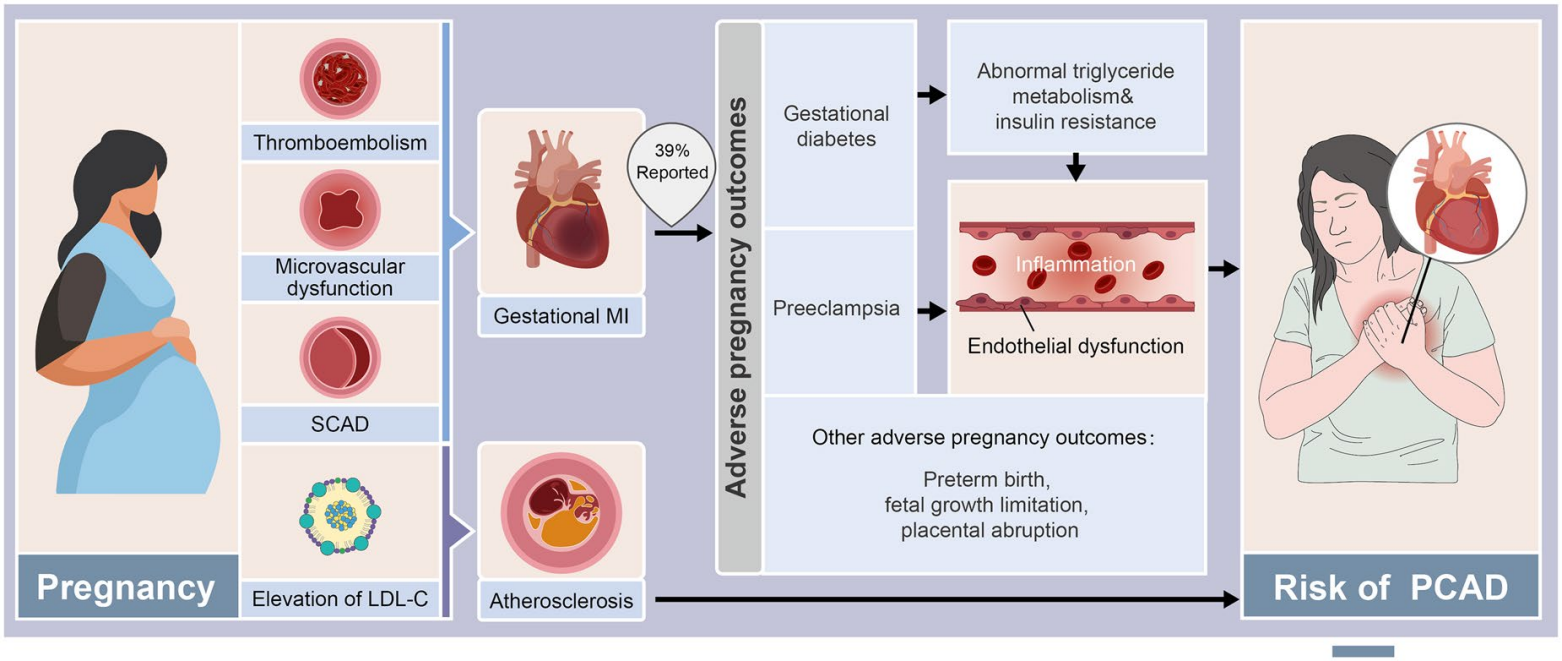
Adverse pregnancy outcomes as risk factors for pregnancy-associated MI and future PCAD. Pregnancy-associated MI is primarily caused by thromboembolism, microvascular dysfunction, or SCAD. Notably, 39% of women with early MI report a history of adverse pregnancy outcomes (e.g. gestational diabetes, preeclampsia). These outcomes are linked to persistent endothelial dysfunction, inflammation, and abnormal metabolism, which drive microvascular dysfunction and atherosclerosis, elevating long-term PCAD risk. Pregnancy-induced LDL-C elevation may accelerate this process.

Placental-mediated issues, including low birth weight (adjusted OR = 2.44), preterm birth (adjusted OR = 2.46), and active gestational asthma (adjusted OR = 3.52), are more common in PCAD women, according to cohort studies [79]. These issues may act as sentinel indications for recurrent PCAD. 39% of women with early MI report adverse pregnancy outcomes, including preterm birth, foetal growth limitation, placental abruption, gestational diabetes, and preeclampsia [80]. Following preeclampsia, these women experience an earlier start of MI and a higher prevalence of diabetes. Preeclampsia triggers this risk by inducing systemic vascular dysfunction, which causes both acute end-organ damage and long-term cardiovascular susceptibility [81,82]. Specifically, a pro-inflammatory state promotes cardiac remodelling (including myocardial hypertrophy, fibrosis, and reduced compliance) [83,84], while endothelial dysfunction exacerbates vasoconstriction, thrombosis, and persistent inflammation [85,86]. Together, these mechanisms establish a sustained state of vascular and metabolic dysregulation, thereby accelerating the later development of coronary artery disease and insulin resistance. Gestational diabetes mellitus (GDM) significantly raises the risk of type 2 diabetes, hypertension, dyslipidaemia, and ultimately fatal ischemic heart disease [87–90]. These include epigenetic modifications, increased inflammatory markers (CRP, IL-6), and carotid intima-media thickness caused by endothelial dysfunction [91,92]. Emerging data suggest that in normoglycemic women with a history of GDM, the TG/Glucose Index exhibits sensitivity similar to HOMA-IR in predicting coronary microvascular dysfunction [93], indicating that triglyceride metabolism and insulin resistance play pivotal roles in GDM-associated long-term cardiovascular risk. Furthermore, a history of GDM is independently associated with damage to the coronary microvascular bed [94], suggesting mechanisms beyond simple macrovascular atherosclerosis. The degree of gestational dysglycemia is associated with dose-dependent cardiovascular risks [95,96] (Figure 1).

### 3.2. Early menopause & ovarian insufficiency

**Figure 2.**
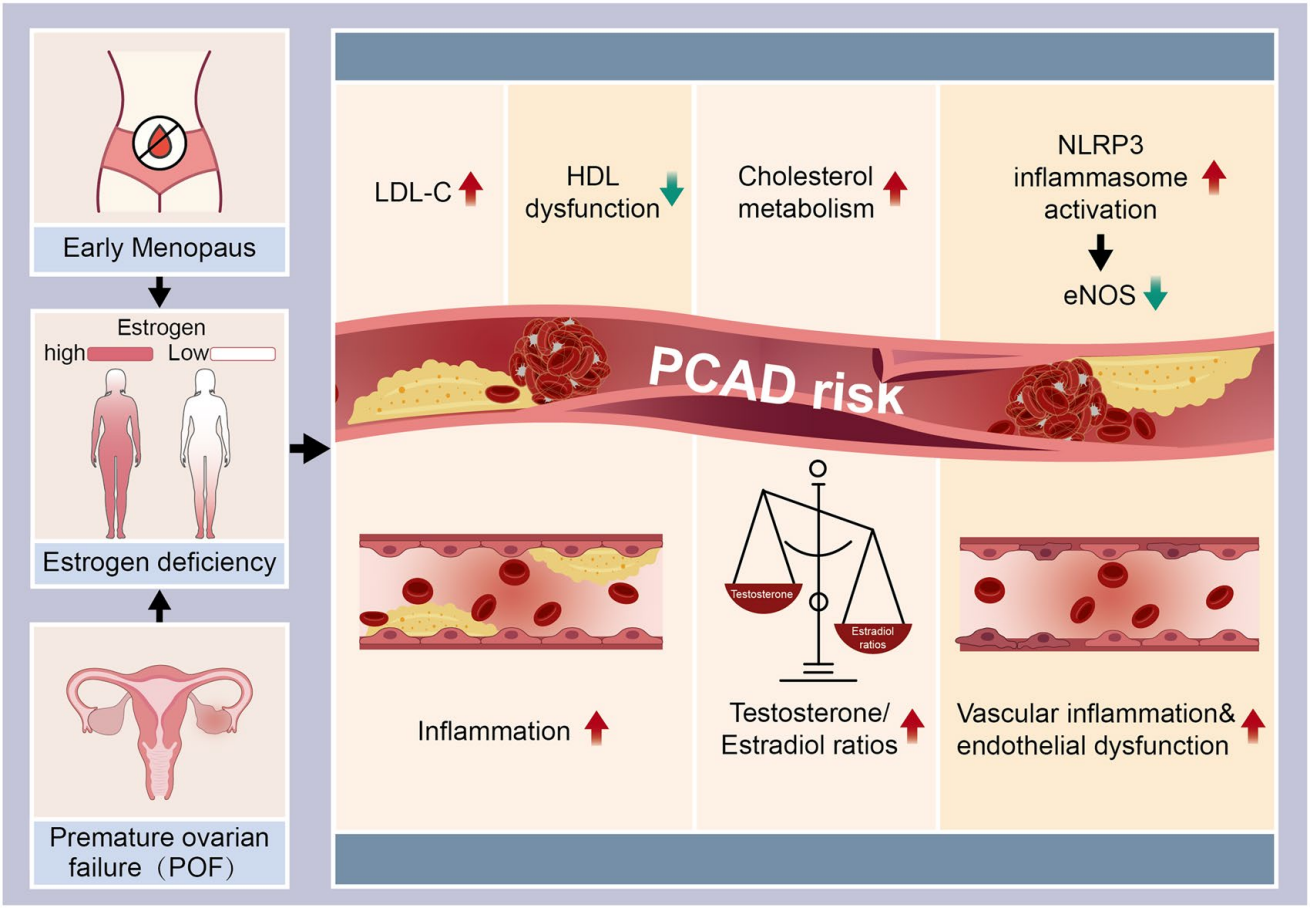
Mechanisms by which premature ovarian failure increases PCAD risk. Early menopause causes chronic oestrogen deficiency, leading to: 1) elevated LDL-C; 2) dysfunctional HDL; 3) elevated cholesterol metabolism; 4) NLRP3 inflammasome activation and eNOS downregulation, exacerbating vascular inflammation and endothelial dysfunction. These changes collectively drive atherosclerosis, significantly increasing PCAD risk.

Young female CAD patients frequently experience premature menopause, which is defined as a permanent cessation of menstruation before the age of 40 and is typically brought on by premature ovarian failure (POF). Menorrhea, vaginal atrophy, and vasomotor symptoms are symptoms of POF, a hypergonadotropic hypogonadism that causes an oestrogen deficiency. While 15% of MI patients under 40 years old reached menopausal status, according to the WAMIF study [65], 9.2% of young MI women reach menopause before the age of 45, according to Chinese population research [33]. On its own, menopause that starts before age 45 increases the risk of MI [97], and meta-analyses have verified associations with increased morbidity and mortality from CVD [98]. An early menopausal transition (MT) and a reduced reproductive lifespan cause chronic oestrogen deficiency, an established risk factor for CVD [99,100]. Oestrogen-mediated vasoregulatory effects counteract chronic inflammation, vascular damage, and long-term RAAS activation associated with early ovarian failure [99,101]. Crucially, oestrogen deficiency caused by early menopause not only increases LDL-C; it significantly impairs HDL functionality. The loss of oestrogen transitions HDL from an ‘anti-atherosclerotic’ to a ‘dysfunctional’ state, compromising its capacity for reverse cholesterol transport and potentially promoting inflammation and accelerating atherosclerosis risk independently of LDL-C levels [102]. Additionally, this hormonal shift removes the inhibition on NLRP3 inflammasome activation and downregulates endothelial nitric oxide synthase (eNOS), thereby directly exacerbating vascular inflammation and endothelial dysfunction [103,104]. Hypoestrogenemia further disrupts cholesterol metabolism, raising testosterone-to-oestradiol ratios and promoting the formation of atherosclerotic plaque, two significant risk factors for heart failure and cardiovascular dysfunction [99,105] (Figure 2).

### 3.3. Fibrinogen

Fibrinogen encourages atherogenesis by causing low-density lipoprotein (LDL) cholesterol to accumulate in arterial walls. Fibrinogen concentrations are elevated in inflammatory situations and are strongly associated with cardiovascular risk factors, including smoking and diabetes. Although acute coronary events are mostly caused by thrombotic processes, fibrinogen’s role in female PCAD is more studied than that of other coagulation factors (t-PA, FVII, FVIII, and PAI-1) [106]. Fibrinogen levels are consistently higher in women than in men, including PCAD groups, even though the underlying causes are yet unknown. Oestrogen has two regulatory effects: it increases fibrinolytic pathways and inhibits the hypertension renin-angiotensin system (RAS) axis. Ironically, IL-6 is released in response to fibrin breakdown products, causing proinflammatory cascades that accelerate the progression of coronary disease. Given that thrombosis, inflammation, and RAS activation work together in concert, young women have more atherogenic potency than men [107].

### 3.4. Disorders of the immune system

Systemic autoimmune diseases like inflammatory bowel disease, systemic lupus erythematosus (SLE) and rheumatoid arthritis (RA) disproportionately affect women. These conditions raise the risk of myocardial infarction, cardiovascular mortality, and premature atherosclerotic cardiovascular disease (ASCVD) [54,55,57,70,108–110]. Women with SLE had a disproportionately higher disease burden than men with the disorder, suggesting sex-dimorphic cardiovascular risks, according to meta-analyses [56]. The Framingham Offspring Study found that young SLE women are more than 50 times more likely to suffer MI than their non-SLE counterparts [58,60]. Anti-phospholipid antibodies specific to SLE promote plaque destabilization, proinflammatory cytokines (TNF-α, IL-1β) cause endothelial dysfunction and lipoprotein infiltration, and altered HDL functionality prevents cholesterol efflux even when HDL-C levels are normal [60,111]. These results are a combination of conventional risk factors and distinct autoimmune mechanisms.

### 3.5. Depression and anxiety

**Figure 3.**
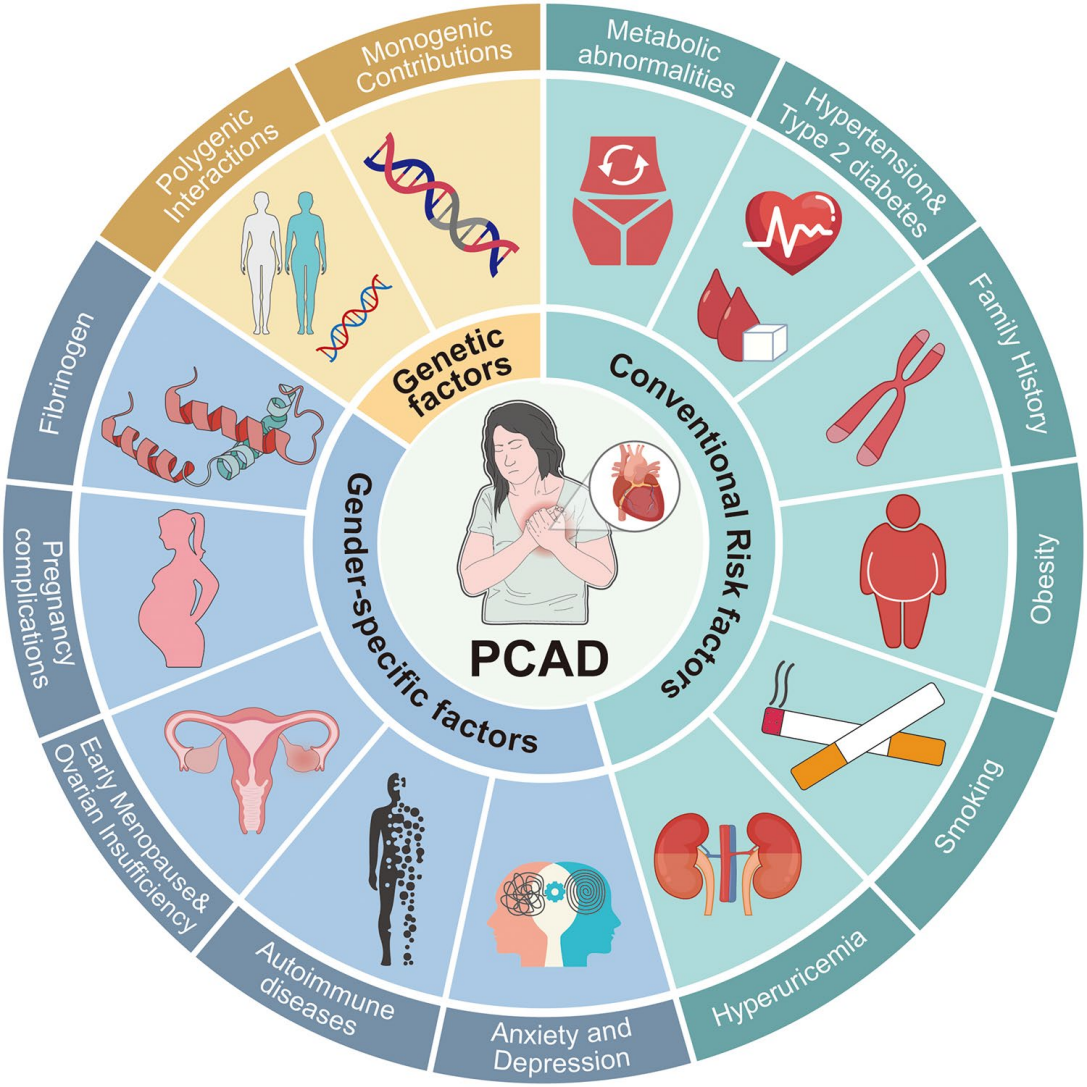
PCAD is more likely to affect women due to a combination of sex-specific problems (pregnancy difficulties, early menopause, autoimmune diseases), metabolic disorders (hypertension, diabetes, obesity), and genetic factors (familial hypercholesterolemia, 9p21 locus). Stronger associations with metabolic syndrome, hyperuricemia, and smoking-related vascular damage are among the main gender differences. A loss of oestrogen exacerbates inflammation and lipid metabolism, and depression and autoimmune disorders like SLE increases risk. These results emphasize the necessity of sex-specific approaches to cardiovascular care, prevention and treatment.

Numerous behavioural, lifestyle, and psychosocial factors that disproportionately impact women and are significant risk factors for cardiovascular illnesses with an early start have also been investigated. There is strong evidence that psychological discomfort is more common in women with early-onset coronary heart disease [112,113]. In addition to being an independent risk factor for myocardial infarction (attributable risk 9%), depression is twice as common in women as in males [57,60,114]. Crucially, this association is mediated through distinct physiological mechanisms rather than serving merely as a behavioural comorbidity. Chronic psychological stress dysregulates the hypothalamic-pituitary-adrenal (HPA) axis, leading to the chronic stress-cortisol-inflammation axis [115]. Persistent hypercortisolemia promotes insulin resistance, visceral adiposity, and systemic inflammation, thereby accelerating atherogenesis [116]. Conversely, acute emotional stress can trigger a massive sympathetic nervous system surge. The resulting catecholamine release might precipitate coronary vasospasm or stress-induced cardiomyopathy (Takotsubo syndrome), providing a mechanistic basis for the vasospastic components frequently observed in female PCAD [117]. An additional follow-up study of patients who had undergone coronary artery bypass grafts revealed that fear and anxiety are linked to being younger than 65 [118]. According to research, women with CAD who are ≤55 years old exhibited more depressive symptoms than men of the same age, and those who are depressed have far higher comorbidities and worse prognoses [119]. 8 years after receiving a heart disease diagnosis, women with PCAD have more anxiety and depression symptoms and have more unfavourable attitudes toward the condition than males [120]. A history of physical and sexual abuse, psychological stress, and post-traumatic stress disorder are among the other psychosocial factors that are currently more inherent to women and are thought to be important risk factors for cardiovascular illnesses [60,110]. The relationship between anxiety and postpartum depression in women of reproductive age and the risk of cardiovascular diseases in the future has not been thoroughly examined and warrants more research [109] (Figure 3).

## 4. Clinical and pathological characteristics

### 4.1. Clinical features

Female PCAD patients typically experience an earlier onset of illness and prolonged clinical courses that span perimenopausal periods. The low sensitivity and specificity of non-invasive diagnostic methods mean that non-specific complaints, including hidden chest pain, are frequently included in their clinical presentations. Sociological research indicates that this group has more psychological comorbidities (anxiety/depression) [27], which are exacerbated by diagnostic challenges when they coexist with perimenopausal symptoms. Misdiagnosis and misinterpretation result from this. Surprisingly, 65% of female PCAD patients do not show the typical symptoms of angina at the time of their initial hospitalization [28].

According to several studies (WAMIF, YOUNG-MI, and VIRGO), chest pain is still the most prevalent AMI symptom in young women (incidence 87%–90.6%) [31,34,121]. However, unusual symptoms such as exhaustion, nausea, vomiting, palpitations, and shortness of breath are more common in patients [34,122,123]. Atypical chest discomfort and overall weakness are among the non-cardiac clinical symptoms that around 20% of very young AMI patients initially present [124]. The diagnosis of AMI requires attention since young patients without cardiac symptoms may cause a delay in the diagnosis.

### 4.2. Coronary lesion characteristics

A five-fold higher prevalence of high-risk plaque (HRP) features, including spotty calcification, positive remodelling, low-attenuation plaques, and napkin-ring signs, is seen on coronary computed tomography angiography (CCTA) in PCAD patients compared to non-CAD controls. This finding is particularly true in cases of recurrent ischemia [125]. In contrast to multivessel involvement, which is more prevalent in older populations, female PCAD patients are more likely to have single-vessel disease (38%–58%), according to angiographic patterns that reveal differences by sex [13,34]. Younger age is significantly associated with isolated vascular disease; patients under 35 years old were 67% more likely to have single-vessel disease than three-vessel involvement, which is rare (8%–14%). The left anterior descending artery (LAD) is the arterial structure most frequently affected, while left main (LM) lesions are still uncommon [126–129]. Young women are more likely to have single-vessel disease and non-obstructive lesions in clinical cases of chest pain [13,34], and they are also more likely to have myocardial infarction with non-obstructive coronary arteries (MINOCA) [130].

#### 4.2.1. SCAD

**Figure 4.**
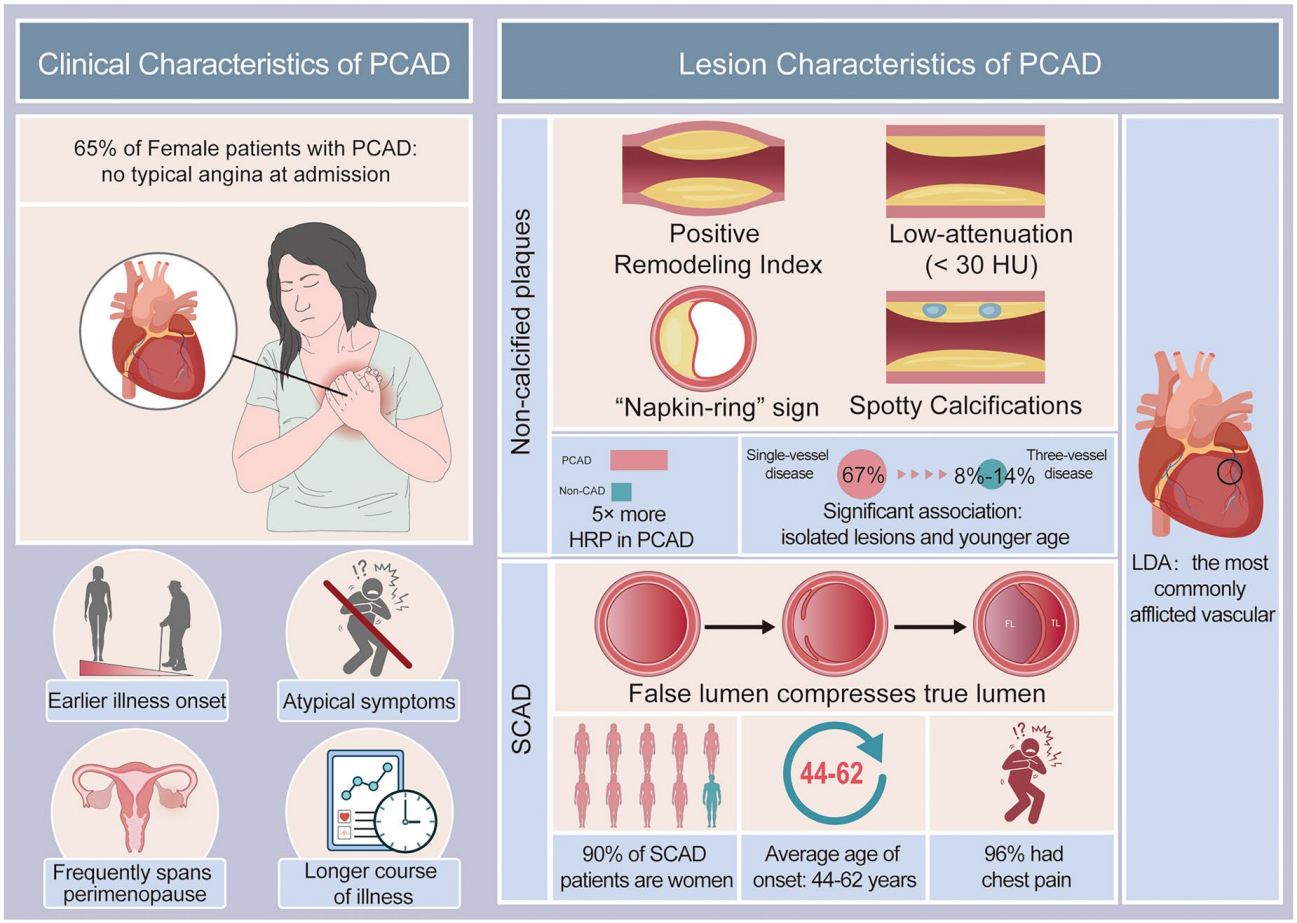
Female PCAD patients frequently have atypical symptoms, a protracted disease, and an early beginning, which frequently results in misdiagnosis. There are many high-risk plaque characteristics. Non-obstructive lesions and single-vessel disease are prevalent, particularly MINOCA. SCAD, which is associated with pregnancy and hormonal changes, is a major cause of AMI in young women.

The U.S. YOUNG-MI study indicates that the prevalence of myocardial infarction with non-obstructive coronary arteries (MINOCA) is much higher in young women, who have an average age of 44 ± 5.1 years, than in men (10.2% vs. 4.2%, *p* < 0.001) [34]. Misdiagnosis of SCAD is common. Due to its overlapping presentations in young populations, atherosclerotic PCAD is characterized by the spontaneous separation of the coronary wall layer, which creates a false lumen that compresses the true lumen and significantly raises the risk of myocardial infarction [131,132]. 90% of SCAD cases occur in women (mean onset age 44–62 years) [133,134], and 96% of cases present with chest discomfort [135]. 35% of cases of acute coronary syndrome (ACS) in women under 50 are caused by SCAD [132,136,137]. Oestrogen increases HDL elevation and nitric oxide-mediated vasodilation, but its abrupt decline following menstruation or pregnancy may increase the risk of SCAD [138–140]. The vulnerability of the arterial wall may also be caused by hormonal changes. Pregnancy-associated SCAD, which mainly occurs postpartum (70% within the first week), complicates 14.5%–43.0% of AMI cases [141,142]. Lesions most frequently affect the left anterior descending artery (50%) and then the circumflex and right coronary arteries [143]. 10% to 30% of SCAD cases recur, particularly in hypertensive patients who can benefit from β-blockers [132,134,144] (Figure 4).

## 5. Therapy management

Patients with premature ASCVD require strict secondary prevention and early screening given their poor prognoses and rapid progression to multivessel disease. management of risk factors. Reducing gender-specific risk factors, controlling diabetes, hypertension and dyslipidaemia, and smoking cessation are still important for people aged 20 to 39. Triglycerides, lipoprotein (a), and apolipoprotein B are components of the non-fasting lipid profile that can identify high-risk patients [145].

Just 40% of patients in secondary prevention receive high-intensity statin medication, and the beginning rate is considerably lower for female and younger patients (less than 50 years old) [146]. Patients with early-onset ASCVD also had poor treatment adherence and limited aspirin/statin drug utilization [147]. Enhanced management of risk variables and treatment compliance are recognized obstacles in secondary prevention. Only 23% of patients with early-onset ASCVD now achieve the LDL-C goal value that is advised by guidelines (<70 mg/dL), and the primary limiting factors are low prescription rates of high-intensity statins and inadequate adherence [148]. By enhancing dyslipidaemia and glycaemic management, PCSK9 inhibitors and novel antidiabetic medications may lower the incidence of major adverse cardiovascular events (MACE) and death through more active secondary prevention [149,150]. Given the prominent role of TRLs and Lp(a) in female PCAD [151], therapeutic strategies must extend beyond achieving LDL-C targets alone. For hypertriglyceridemia, high-dose icosapent ethyl (EPA) has been proven to reduce cardiovascular event risks [152]. Furthermore, novel inhibitors targeting ApoC3 show promise in lowering TRLs. For elevated Lp(a), PCSK9 inhibitors can cause a modest suppressive effect, while more targeted antisense oligonucleotide therapies have made breakthrough progress in clinical trials [153]. Incorporating these emerging therapies into the comprehensive management of female PCAD is a vital future direction. The advantages of a Mediterranean diet and consistent exercise have been amply demonstrated by research, even in the face of low adherence to lifestyle interventions [154].

**Figure 5.**
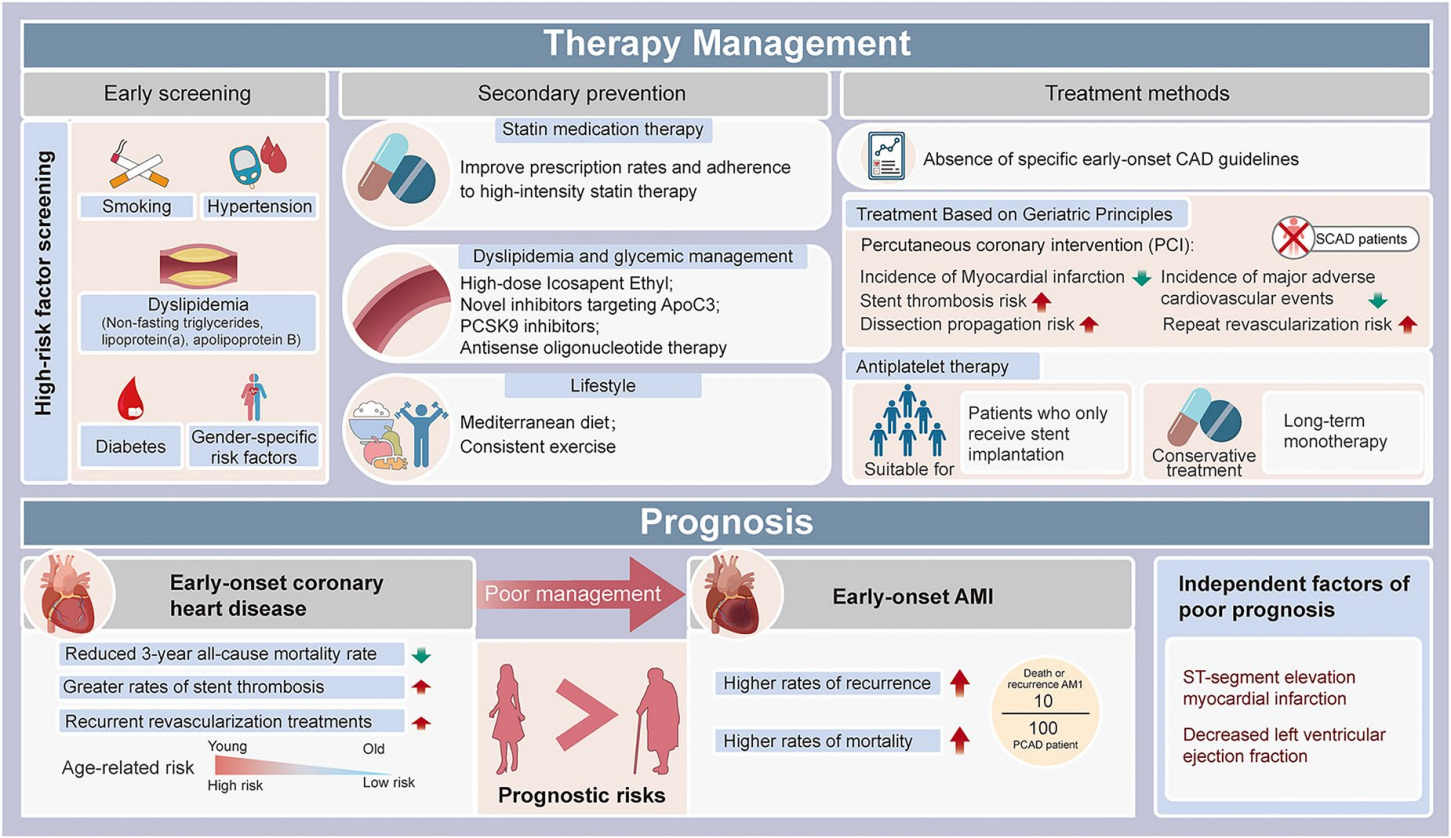
Comprehensive management and prognosis characteristics of patients with PCAD. Therapy Management: Emphasizing early screening (smoking, hypertension, dyslipidaemia, diabetes, and gender-specific factors) and intensive secondary prevention. Current treatment faces three key challenges: absence of dedicated guidelines (often following protocols for elderly patients), low utilization and adherence to high-intensity statins, and the need to integrate novel therapies like PCSK9 inhibitors and ApoC3-targeting agents for optimal lipid management. Percutaneous coronary intervention (PCI) is underutilized in young patients and carries higher risks of stent thrombosis and repeat revascularization. Prognosis: while PCAD patients show lower 3-year all-cause mortality, they face significantly higher risks of stent thrombosis and re-intervention. Early-onset AMI (particularly in women) is associated with elevated recurrence and mortality rates, with ST-segment elevation myocardial infarction and decreased left ventricular ejection fraction serving as independent predictors of poor outcomes. Overall, inadequate management of modifiable risk factors constitutes the core issue driving adverse prognosis, highlighting the imperative for enhanced preventive strategies.

Currently, PCAD patients are typically treated without special guidelines using the same medications as elderly individuals, such as statins, β-blockers, ACEI/ARBs, and dual antiplatelet therapy (DAPT). However, according to a Norwegian study [13], compared to the 45–60 age range, individuals under 45 had considerably lower rates of coronary angiography (88% vs. 91%) and percutaneous coronary intervention (PCI, 68% vs. 73%) (*p* < 0.001). The same group of patients had a higher DAPT utilization rate (89% versus 80%; *p* < 0.001) than patients aged 60–80. Compared to men, young women are less likely to have PCI, DAPT, and statin therapy [6,34]. According to two other investigations, young female AMI patients have a lower PCI rate than their male counterparts of the same age [155,156]. Patients with initial myocardial infarction and PCAD had a lower all-cause death rate within three years following PCI than patients with non-early-onset CAD in four large-scale drug-eluting stent trials [7]. PCI has a higher risk of stent thrombosis and repeat revascularization, even though it can lower the incidence of MACE in patients with myocardial infarction and early-onset coronary heart disease [157]. Crucially, the management of SCAD is entirely different from that of atherosclerotic PCAD. Initial management of SCAD is generally conservative. Unless there is ongoing ischemia or hemodynamic instability, PCI should be avoided as it might lead to dissection propagation. β-blockers are the cornerstone of therapy, reducing vascular shear stress and the risk of recurrence. Regarding antiplatelet therapy, current guidelines recommend a more cautious approach; short-term dual antiplatelet therapy (DAPT) is reserved for patients receiving stents, while long-term monotherapy may be the optimal choice for those managed conservatively [158–160]. Consequently, controlling modifiable risk factors, such as blood sugar, blood lipids, smoking, and weight, must be given top priority (Figure 5).

## 6. Prognosis

According to certain research, PCAD patients have a lower rate of adverse events [13,161]. PCAD patients have a reduced 3-year all-cause mortality rate, but they also have greater rates of stent thrombosis and recurrent revascularization treatments. Younger patients have lower rates of MACE, all-cause mortality, and cardiac mortality when comparing men and women by age percentiles [157]. However, early-onset AMI has significantly higher rates of recurrence and mortality, especially in women and patients with obstructive lesions, since modifiable risk factors are not adequately managed [32,34]. With roughly 10 events per 100 patients per year, including mortality or recurrent myocardial infarction, the prognosis is significantly worse than in older high-risk populations [162]. Women are not given the appropriate prophylactic measures because of the false belief that they do not exhibit the typical symptoms of atherosclerotic thrombosis.

ST-segment elevation myocardial infarction and decreased left ventricular ejection fraction are both independent predictors of a poor outcome [163]. Acute myocardial infarction raises the in-hospital death rates in patients with cardiopulmonary arrest (CPA), which is more common in younger people, according to a study by Ando et al. [164]. This poor prognosis underscores the need for more aggressive primary and secondary prevention, whether by medication to help smoking cessation and lower LDL-C or lifestyle changes to lose weight and maintain a healthy diet [32]. The differences in the pathophysiology of AMI between young and elderly individuals must also be considered. Even though plaque rupture is the main cause in both cohorts, the younger age group is more likely to experience plaque erosion, spontaneous coronary artery dissection, and vasospastic angina. The difference in in-hospital death rates between the two groups may be caused in part by this [165] (Figure 5).

## 7. Research problems and opinions

Despite recent significant improvements in diagnosis and treatment, about 20% of PCAD patients cannot be explained by traditional atherosclerosis mechanisms, suggesting that there are still significant gaps in the disease’s pathological mechanisms [25]. Notably, PCAD is increasingly prevalent in women, and the condition is made even more complex by the combination of metabolic inflammation and specific gender-related risk factors, including autoimmune diseases, early menopause, and pregnancy problems. Early screening instruments and focused prevention and treatment strategies are therefore necessary [166]. Young female PCAD patients frequently have atypical clinical symptoms, non-obstructive coronary artery lesions, and a high rate of SCAD, which may lead to a delayed diagnosis and inadequate treatment. Inadequate lipid control, gender-biased clinical intervention strategies, and poor secondary preventive adherence all raise the likelihood of a poor prognosis.

To improve prognosis, clinical paradigms must shift from a generic approach to phenotype-specific strategies. This includes strictly differentiating the conservative management of SCAD from the aggressive lipid-modifying therapies required for atherosclerotic PCAD (targeting not just LDL-C but also TRLs and Lp(a)). Future research and guidelines should prioritize: 1. Using sex-specific biomarkers and multi-omics to detect problems early; 2. Translating the identified pathophysiological bridges, such as the oestrogen-ABCG2 axis and inflammatory cascades, into targeted therapeutic interventions; 3. Creating clinical guidelines that are gender-stratified; 4. Using digital health tools to increase treatment compliance.

## Data Availability

Data sharing is not applicable to this article as no data were created or analysed in this study.
